# Polymer-Based Electrospun Nanofibers for Biomedical Applications

**DOI:** 10.3390/nano8040259

**Published:** 2018-04-20

**Authors:** Abdullah M. Al-Enizi, Moustafa M. Zagho, Ahmed A. Elzatahry

**Affiliations:** 1 Department of Chemistry, King Saud University, P.O. Box 2455, Riyadh 11451, Saudi Arabia; amenizi@ksu.edu.sa; 2 Materials Science and Technology Program, College of Arts and Sciences, Qatar University, P.O. Box 2713, Doha, Qatar; mmsalah@qu.edu.qa

**Keywords:** electrospinning, nanofibers, medical prostheses, wound dressing, drug release, tissue engineering, blood vessels, bone

## Abstract

Electrospinning has been considered a promising and novel procedure to fabricate polymer nanofibers due to its simplicity, cost effectiveness, and high production rate, making this technique highly relevant for both industry and academia. It is used to fabricate non-woven fibers with unique characteristics such as high permeability, stability, porosity, surface area to volume ratio, ease of functionalization, and excellent mechanical performance. Nanofibers can be synthesized and tailored to suit a wide range of applications including energy, biotechnology, healthcare, and environmental engineering. A comprehensive outlook on the recent developments, and the influence of electrospinning on biomedical uses such as wound dressing, drug release, and tissue engineering, has been presented. Concerns regarding the procedural restrictions and research contests are addressed, in addition to providing insights about the future of this fabrication technique in the biomedical field.

## 1. Introduction

The decline in the size/diameter of the polymer fiber from micrometers to nanometers has many advantages such as increased surface area and surface functionality [1]. Polymer nanofibers exhibit enhanced characteristics for several applications [2]; fabricated via self-assembly process [3,4], phase separation [5], and electrospinning [6,7]. Electrospinning has been applied in the recent years to fabricate polymer nanofibers. Utilizing the template synthesis technique, nanoporous membranes can be produced. The main advantage of this technique lies in its ability to fabricate nanometer scale tubules and fibrils of different materials such as electronically conducting carbon, metals, polymers, and semiconductors [8]. In contrast, the non-ability to produce one-by-one continuous nanofibers can be disadvantageous [9]. The self-assembly method is a time-consuming technique in which individual, pre-existing components can organize themselves, yielding nanostructural morphology [3,4]. The nanoscale porous foam can also be produced by the phase separation process consisting of multiple steps such as extraction, gelation, and dissolution using different solvent, drying, and freezing techniques [1]. However, similar to the self-assembly method, this technique also takes a comparatively long time. The structure and diameter of the electrospun nanofibers can be controlled by various aspects, and classified into ambient, process, and solution parameters. Thus, the electrospinning technique is considered as an important candidate for a mass fabrication strategy of one-by-one continuous nanofibers in which the process can be optimized for different polymer solutions [1].

### Electrospinning Process

Electrospinning main setup consists of a high-voltage power supply connected to a spinneret and a grounded collector operating under ambient conditions at room temperature. The solution is charged when a sufficiently high voltage is applied to the solution droplet at the tip of spinneret, leading to electrostatic repulsion, droplet stretching, and accelerated towards the collector of opposite polarity [10]. The solvent evaporates, leaving polymer nanofibers [11]. Currently, there are two main standard setups for the electrospinning technique: vertical and horizontal. It is well known that the electrospinning technique has many disadvantages [12]. First, in the fabrication of organic nanofibers, the types of polymers used are limited and the behavior of nanofibers is not well discussed. In addition, inorganic nanofibers have been limited owing to their friability after the calcination process. The high-cost production of electrospun nanofibers with a large diameter represents a significant challenge in nanofiber fabrication.

## 2. Applications of Nanofibers

It is remarkable that nanofibers can be applied in a wide range of industries such as semiconductors [1], protective materials (chemical resistant cosmetics and sound absorption) [1], water purification [13], and clean energy applications [13]. The most promising applications lie in the biomedical field, which includes drug delivery carriers, tissue engineering, and wound dressing [1]. It is noteworthy to report that the electrospinning process can be applied to prepare metal oxide nanofibers for a wide range of applications [14]. There are still serious limitations regarding the improvement of functional nanofibers and reproducible electrospinning processes [13]. Therefore, there is growing interest for the development of efficient, stable, and versatile electrospinning process tools. Several companies are aiming to fulfill these requirements by providing user-friendly, unique, reliable, and environmentally friendly electrospinning equipment.

### 2.1. Biomedical Applications

Most of human organs and tissues such as bone, collagen, dentin, and skin are present in a nanofibrous form. They are characterized by organized hierarchical fibrous structures that realign in nanometer scale, motivating and steering most of the nanofiber research towards biomedical and bioengineering applications [15]. There are unusual physical properties of fibers and fiber mats such as surface area, diameter, and porosity, which resemble similar characteristics to the extracellular matrix (ECM). This novel morphology enhances cell behavior by segregating tissues from one another and offering promising anchorage and support for cells [15,16,17]. Characteristics of the ECM may be especially appropriate for the functional characteristics of biomaterials. Fiber mats are used to control cell proliferation, migration, and other cell tissue aspects [15,17,18,19]. Nanofibrous materials are also used in other biomedical applications such as medical implants, wound dressings, antimicrobial agents, drug delivery vehicles, biomimetic actuators, dental materials, enzyme immobilization scaffolds, and protective textiles for chemical and biological threats [10,20,21]. Biocompatible and biodegradable polymers are required to fabricate nanofibers used in biomedical application. The polymers may also have to be treated with bioactive cell-organization ligands to perform certain functions [15]. Functionalization was found to reduce the inflammatory activation of T cells, mast cells, and macrophages with the release of cytokines [22]. For tissue engineering approaches, the nanofiber scaffolds with seeded cells are incorporated [23]. For wound healing, the porous structure helps drug particles diffuse out of the matrix more efficiently [24]. The drug release rate can be measured by controlling the thickness of the synthesized nanofibrous mat [24]. For drug delivery systems, the nanofibrous membranes were implemented with the drug component to deliver the targeted drug to the human body [1].

In the following sections of this review, aforesaid promising biomedical advances of electrospun nanofibers are discussed, and the research concept of each approach is briefly addressed.

#### 2.1.1. Medical Prostheses

Electrospun fibers exhibited high potential in prosthetic devices used in surgical operations. The soft texture and the nature of prepared fibers made them excellent candidates to be utilized as a coating for hard tissue prosthetic devices. This coating sheet with fibrous morphology inhibits the device failure by diminishing the stiffness mismatch at the tissue/device interphase by acting as interphase between the host tissues and the prosthetic system [1,25]. It has also been reported that nanofibrous materials have been selected as unique candidates in a wide range of tissue prostheses including breast, vascular, blood vessel, etc. [1,25,26,27,28,29,30]. It was reported that nanofiber coating prevents infections of prosthetic joints [31]. In addition, Popryadukhin et al. fabricated vascular prostheses based on nanofibers from copolymer of ε–caprolactam and hexamethylendiaminadipate [32]. Recently, for liver tissue engineering, Semnani et al. [33] fabricated a nanofibrous scaffold from polycaprolactone (PCL) and chitosan (CS) using a novel collector to make improved pore size and orientation for cell infiltration.

#### 2.1.2. Wound Dressing

**Figure 1:**
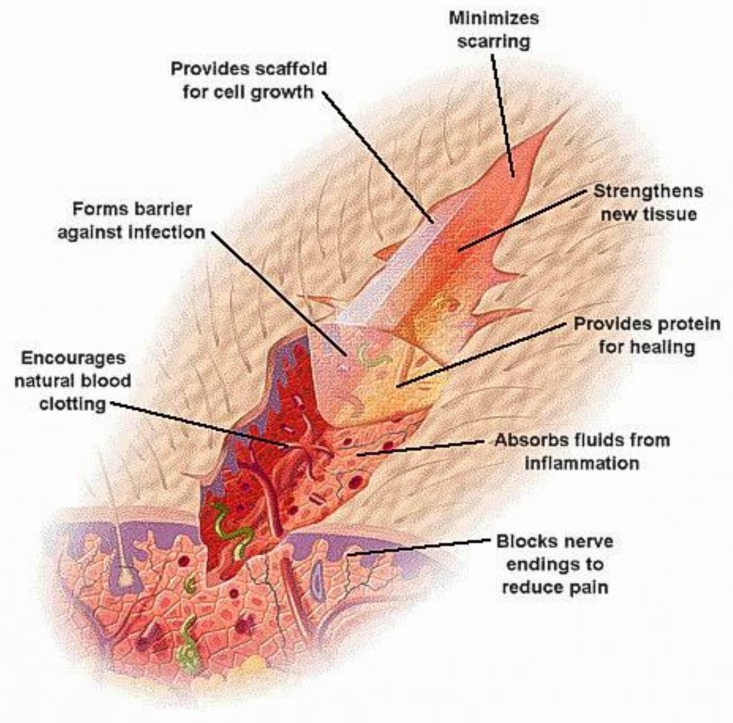
An illustrative diagram of the desired characteristics of wound dressing products. Reproduced with permission from [38,39]. Elsevier, 2011.

Wound dressing represents a significant issue to be dealt with in the biomedical field. The warm, nutritious, and moist environment offered by wound beds offers a perfect condition for microbial growth [34,35]. Excellent antimicrobial dressings should exhibit good broad-spectrum antimicrobial behavior, provision of a moist environment, gas permeation, and performance against antibiotic-resistant bacteria to improve healing processes (Figure 1) [36]. Consequently, to avoid microbial infection, trans-epidermal water loss leading to an acceleration of wound regeneration [34] and quick care of skin wounds are required [37]. Therefore, skin barrier restoration is very effective to treat injuries.

The recent reports of nanotechnology fields discussed the synthesis processes of nanofibrous conducts exhibiting the same structural and architectural features of natural ECM [40]. For this approach, the natural ECM is replaced by nanofibers until the host cells can grow, and new ECM will be produced. Nanofiber mats can rapidly initiate signaling pathway and draw fibroblasts to the dermal layer, which provide many cytokines and collagen [34]. Polymer nanofibers have been used extensively for wound treatment application. The used nanofibers exhibited pore size ranging from 500 nm to 1 mm and high surface area. The electrospun biodegradable polymer nanofibers can be sprayed onto the damaged skin to produce a fibrous dressing to protect it from bacterial infection via aerosol particle capturing pathways. This technique has been used to treat skin problems such as wounds and burns. This will help the wounds to heal by forming normal skin growth and reducing the scar tissue that would develop otherwise in the conventional treatments [1,41]. Researchers focused on fabricating enhanced wound dressings from biocompatible materials [39,42,43,44]. Current reports discuss the usage of biologically derived materials such as chitin to synthesize accelerated repair wounds.

In 2011, Jayakumar et al. [45] published a review article describing the importance of electrospun chitin and chitosan nanofibers as novel wound dressings for healing [39]. It has been mentioned that electrospun chitin and its derivative nanofibers provide unique characteristics, which will lead to accelerating wound healing such as liquid absorption, high durability, low toxicity, antibacterial activity, and good biocompatibility. Chilarski et al. [46] reported that fibrous materials made of dibutyrylchitin (DBC) were used for wound healing applications. Using a group of several patients, they presented a satisfactory data of wound healing in many uses, particularly in postoperative/posttraumatic and burn wounds [46]. On the other hand, Blasinska and Drobnik studied the repair processes and its mechanisms of action using DBC [47]. It was noted that the weight of the granulation tissue of rats was surged by DBC implanted subcutaneously. Also, it was found that the number of cells separated from the wounds and cultured on the DBC films increased. This report describes the impact of DBC on the number of cells and extracellular matrix [47]. Also, Zhou et al. prepared biocompatible carboxyethyl chitosan/poly(vinyl alcohol) (CECS/PVA) nanofibers for wound dressing applications [48]. In vitro measurements using mouse fibroblasts (L929) were employed to evaluate the fabricated CECS/PVA fibrous mats as scaffolds templates for skin regeneration. The promotion of L929 cell attachment and proliferation was improved by these nanofibers [48]. On the other hand, Ignatova et al. [49] reported that electrospun mat made of quaternary chitosan (QCS) is a promising material for wound dressing applications and actively inhibited the growth of Gram-positive and Gram-negative bacteria [49,50]. Also, biocompatible, biodegradable composite of electrospun alginate/chitin nanofibers has been prepared and used as wound dressing material [45,51]. The small contents of chitin nanofibrils were noticed to enhance the susceptibility to lysozyme. Also, the release of chitin oligomers affects the efficiency of calcium-alginate wounds dressing. Another biocompatible composite based on chitosan/collagen of durable sandwich wounds dressing with excellent antibacterial behavior and high liquid absorption was fabricated [52]. In this study, different weight ratios of chitosan to collagen were applied to immobilize on the polypropylene nonwoven fabric, which was pre-grafted with *N*-isopropyl acrylamide (NIPAAm) or acrylic acid (AA) [52]. It was investigated that NIPAAm-grafted and PP-NIPAAm-collagen-chitosan exhibited better healing impact than AA-grafted and PP-AA-collagen-chitosan. The wounds treated with PP-NIPAAm-collagen-chitosan offered high remolding impact in histological testing compared to the construction of epidermis at 21 days after skin injury. On this aspect, Chen et al. [53] reported the preparation of composite nanofibrous membrane. In this report, electrospinning of polyethylene, chitosan, and type 1 collagen was proceeded to prepare the nanofibers and then followed by cross linking using glutaraldehyde vapor. The prepared nanofiber exhibited a diameter of 134 ± 42 nm, which enlarged to 398 ± 67 nm after crosslinking. It was found that Young’s modulus enlarged after crosslinking, while water sorption capability, tensile strain, and tensile strength declined. Prepared nanofibers displayed excellent in vitro biocompatibility and no cytotoxicity toward the development of 3T3 fibroblasts [53]. Another antibacterial wound dressing electrospun fiber prepared from sodium alginate (SA)/poly (vinyl alcohol) (PVA) was reported by Shalumon et al. [54]. In this work, adding ZnO particles increased the diameter of the produced fibers. Also, antibacterial measurements revealed that the fabricated mats for all ZnO loadings offer inhibition in both bacteria strains, and, as the content of ZnO increases, the inhibition increases [54].

Kossovich et al. [55] proposed the preparation of chitosan-based electrospun nanofibers for burn wound dressing. Burn healing is considered as one of the most critical complications of modern surgery owing to high disability and lethality after burns treatment. The prepared wound dressings were tested for IIIa and IIIb degree burns. These nanofiber dressings exhibited adequate wound ventilation, exudate absorption, and protection from infections [45,55]. In another study, silk fibroin and chitosan composite nanofibrous membranes were synthesized by electrospinning [56]. In this work, the prepared electrospun fibers were tested against *E. coli* (Gram-negative) and *S. aureus* (Gram-positive) by turbidity tests. On the other hand, the biocompatibility of murine fibroblast on the synthesized membranes was realized to promote cell proliferation and attachment [45]. Another study achieved by Chong et al. [57] introduced a new low cost composite consisting of a nanofibrous scaffold directly electrospun onto a polyurethane wound dressing. In this study, they determined the feasibility of the TG-NF construct as an effective TE scaffold for wound healing. The study realized that HDF cells could proliferate and develop on the TG-NF construct, as well as on nanofibers scaffolds [57]. Costache et al. [58] reported the preparation of an active composite active for wound dressing applications. It was prepared from two fully tissue compatible and biodegradable materials. Tyrosine-poly(ethylene glycol)-derived poly(ether carbonate) copolymer matrix was embedded by silicon oxide sol-gel microparticles [58].

Unnithan et al. reported the successful preparation of uniform nanofibers of polyurethane–dextran loaded with ciprofloxacin drug. They showed that the addition of the drug reduced the size and narrowed the distribution of electrospun nanofiber diameters due to the decrease in solution viscosity. In addition to that, they proved that the cell viability and attachment were enhanced after incorporating dextran into the polyurethane. The nanofibers provided excellent antibacterial property for both Gram-negative and Gram-positive bacteria [34].

**Figure 2:**
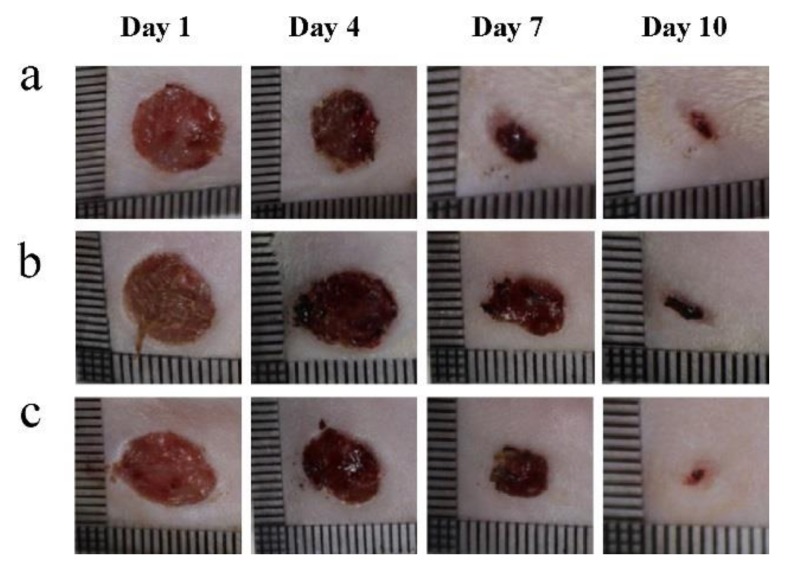
Presence of would healings at 1, 4, 7, and 10 days after adding (**a**) 30% LZ loaded CS–EDTA/PVA nanofiber mats, (**b**) gauze (-ve control), and (**c**) commercial antibacterial gauze dressing (Sofra-tulle^®^) (+ve control). Reproduced with permission from [59]. Elsevier, 2012.

In 2012, several publications related to electrospun fibers for wound dressing applications were published. Chitosan-based nanofiber mats loaded with Lysozme for wound healing were fabricated by Charernsriwilaiwat et al. [59]. In their report, a blend of PVA and chitosan-ethylenediaminetetraacetic acid (CS-EDTA) and lysozyme was electrospun to fabricate mats (average diameter of 143–209 nm). In animal wound healing studies, loaded CS-EDTA mats exhibited an accelerated healing rate compared to the reference (gauze) (Figure 2).

Elzatahry et al. [60] reported that nanofiber composites that introduced N-heterocyclic carbenes were successful as antimicrobial materials. In this study, they electrospun gold acetate or gold chloride complexes of bis(imino)acenaphthene (BIAN)-supported NHC with PVA aqueous solutions. The diameter of the prepared nanofibers ranged from 250 to 300 nm. Two fungal strains, two Gram-positive bacteria, and six Gram-negative bacteria were used to study the biological performance. Prepared nanofibers recorded no sensitivity against the fungal strains. Besides, gold chloride complexes showed interesting activity against all Gram-positive bacteria and one Gram-negative bacteria [60].

The core-shell structure of poly(lactic acid)/chitosan nanofibers was prepared by Li et al. [61]. In this report, L929 cell culture revealed that the nanofibers controlled the cell proliferation and adhesion. These nanofibers are considered as potential candidates for wound dressing and tissue engineering applications. It was shown in this study that electrospinning process of neat chitosan is very hard owing to its poor mechanical performance. On the other hand, poly(lactic acid) was used as core polymer due to its excellent mechanical, biocompatibility, and electrospinning nanofiber-producing capability [61]. A tray to mimic the biological, mechanical, and physical cues offered by the skin dermis was reported by Rnjak-Kovacina et al. [62] through electrospinning of collagen and tropoelastin to combine the unique characteristics of both proteins. They stated that as the collagen content increases, the electrospinning efficiency declines. The prepared scaffolds provided elasticity similar to that of the pure SHE scaffolds. Also, collagen was found to enhance the manipulation and handling of the fabricated scaffolds.

Pant et al. [63] reported the synthesis of nylon-6 nanofibers incorporated with Ag nanoparticles. Methoxy poly(ethylene glycol) and formic acid were used as electrospinning solvents to convert AgNO_3_ to Ag nanoparticles. The study showed that the fabricated Ag/nylon-6 composite nanofibers have isotropic morphology, with Ag nanoparticles dispersed uniformly throughout the polymer galleries. Furthermore, the composites have excellent antibacterial property for biofilm, wound dressing, and filtration uses.

**Figure 3:**
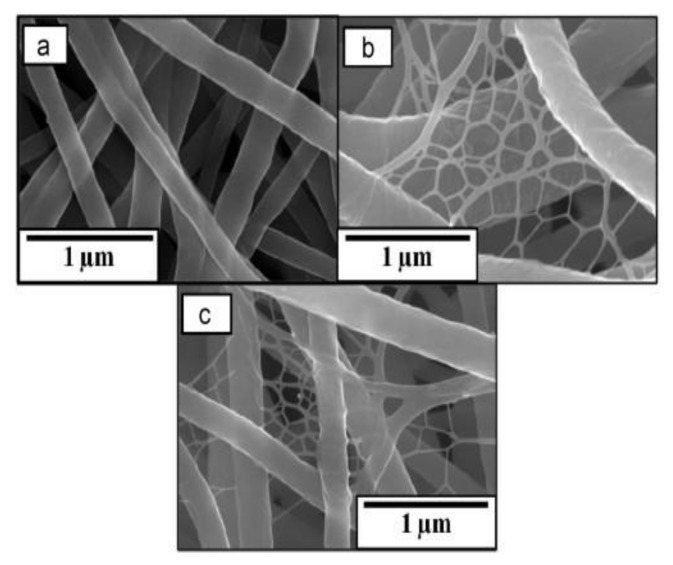
FE-SEM images of (**a**) neat PU, (**b**) ZnO doped PU, and (**c**) ZnO/Ag-doped PU nanofibers. Reproduced with permission from [64]. Elsevier, 2013.

Shamshi Hassan et al. [64] mentioned the synthesis of electrospun nanofibers from Bimetallic ZnO/Ag embedded polyurethane (Figure 3). It was proven that incorporating Ag and ZnO particles into prepared materials exhibited an enhanced bactericidal effect without any harmful influences upon normal mouse fibroblast cells. This behavior confirmed the benefit of using these mats in filtration, clinical, and textile uses [64].

**Figure 4:**
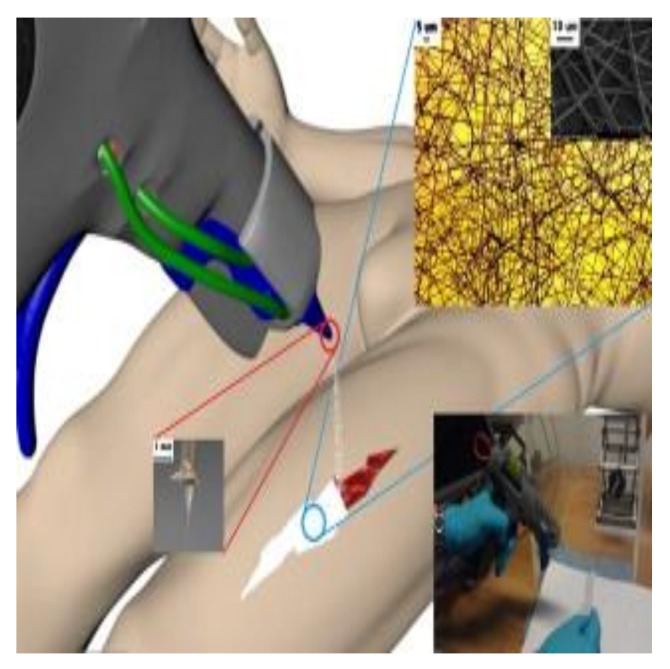
Design and performance of electro hydrodynamic multi-needle spray gun for a wide range of biomedical uses. Reproduced with permission from [65]. Elsevier, 2013.

Sofokleous et al. [65] reported the design and testing of a portable handled electro hydrodynamic multi-needle device. This device was used to fabricate multifunctional fibers (Figure 4). Multifunctional encapsulated fibers with sub-micrometer to micrometer range dimensions were prepared using the spray gun. Rath et al. loaded silver nanoparticles into collagen nanofibers using electrospinning technique for accelerated wound healing [66]. Silver nanoparticles were loaded owing to their high antimicrobial activity. Moreover, collagen acts as a primary morphological candidate for the extracellular network to develop cell growth, adhesion, and differentiation. Antimicrobial and MIC measurements were used to investigate the antibacterial properties of silver nanoparticles and their composite nanofibers. The excellent wound-healing performance of the fabricated mats was revealed by in vivo measurements. This can be attributed to their anti-inflammatory, intrinsic antimicrobial activity, and controlled drug-release behavior [66].

There have been significant advances to enhance the performance of wound dressings. These trends include silver nanoparticles-incorporated nanofibers [67], polymeric nanofibers involving green tea extract [68], gelatin nanofibers loaded with vitamins A and E [69], antimicrobial honey/chitosan nanofibers with Cleome droserifolia and Allium sativum [70], tetracycline hydrochloride-loaded nanofiber mats based on PVA and chitosan [71], and levofloxacin-loaded nanofibers [72]. These concepts improve the healing process by providing mechanical protection, moist environment, and desirable conditions for wound healing. In addition, Jiang et al. fabricated a novel UV protecting nanofibrous membrane using the incorporation of silica nanocapsules loaded with functional payloads into polymer nanofibers for antibacterial wound dressing [73]. Dimethyloxalylglycine-embedded poly(ε-caprolactone) fiber meshes were fabricated by Zhang et al. [74] for wound healing in diabetic rats. These meshes promoted the wound healing by modulating macrophage responses, improving nerve innervation and angiogenesis, and enhancing ECM fabrication [74]. Kandhasamy et al. [75] fabricated collagen-coated ostholamide nanofibers from osthole. These scaffolds displayed an effective antibacterial performance against common wound pathogens, *S. aureus* and *P. aeruginosa* [75]. Recently, Wang et al. [76] synthesized silk fibroin (SF)/GO nanofibers with bioinspired nanomorphology for wound dressing applications. It was found that graphene oxide enhanced the biocompatibility and antibacterial properties of SF nanofibers [76].

#### 2.1.3. Drug Release

It is well known that the amount of drug actually delivered to the target site within the human body is much lower than the initial orally ingested drug dose, as it spreads to other healthy sites through the digestive organs. Hence, patients are required to take excessive amount of medication, causing unfavorable side effects. It has been found that the optimum drug amount is actually the minimum needed content of the drug to the target site and the minimum needed to be absorbed efficiently at the disease site. The rapidity of the uptake of the drug into the body is directly proportional to the size of the drug. Therefore, polymers including micelle and hydrogels were used to improve the drug carriers [77,78]. Although these carriers reduce the side influences and enhance the therapeutic impact, there is still a requirement to discuss the control of the rate of drug release. Based on such experience, scientists are currently engrossed in employing polymer nanofiber drug carriers owing to their novel inherent nanoscale morphological characteristics [78,79]. Moreover, different systems containing drugs can be synthesized from monolithic nanofibers to multiple different hierarchical structures [10]. The mechanism of drug release is measured by both polymer degradation and diffusion processes within nanofiber network. Different polymers, polymer characteristics, and surface coatings can be employed to tailor the performance of drug release [79,80,81,82,83].

Drug-loaded nanofibers can be employed as implants and to aid in targeted drug delivery system. For controlled release, therapeutics such as polysaccharides, antibiotics, and anti-cancer drugs can be loaded into nanofibers [84,85]. Therefore, drug-loaded nanofibers support cellular differentiation and proliferation through simulating the topography of the native ECM [85]. In 2002, Kenawy et al. [86] came out with the first study related to the use of electrospun fiber for drug delivery applications. In their research, for periodontal disease treatment, tetracycline hydrochloride drug was loaded into PLA, poly(ethylene-co-vinyl acetate) (PEVA), and PLA/PEVA blend (50:50) nanofibers. It was reported that the drug content was 5% in each system. On the other hand, the release profile was found to depend on the nature of the used polymer and the amount of loaded drug [86]. Later on in 2003, Verreck et al. [87] studied some of the factors affecting the release of drug from the electrospun fiber, such as the drug content, drug distribution in nanofibers, and distribution of fiber diameter. In this research, the drug was loaded into hydroxylpropylmethyl cellulose (HPMC) nanofiber membranes. Then the membranes were folded and transferred in a hard gelatin capsule. It was revealed that nanofibers filled in capsules released the drug over 20 h, while the neat nanofiber membranes released the drug over 4 h only [78,87]. In general, the simple procedure to develop drug delivery system was mainly based on electrospinning a combination of polymer and drug dissolved in the same solvent [24,84,88,89,90].

**Figure 5:**
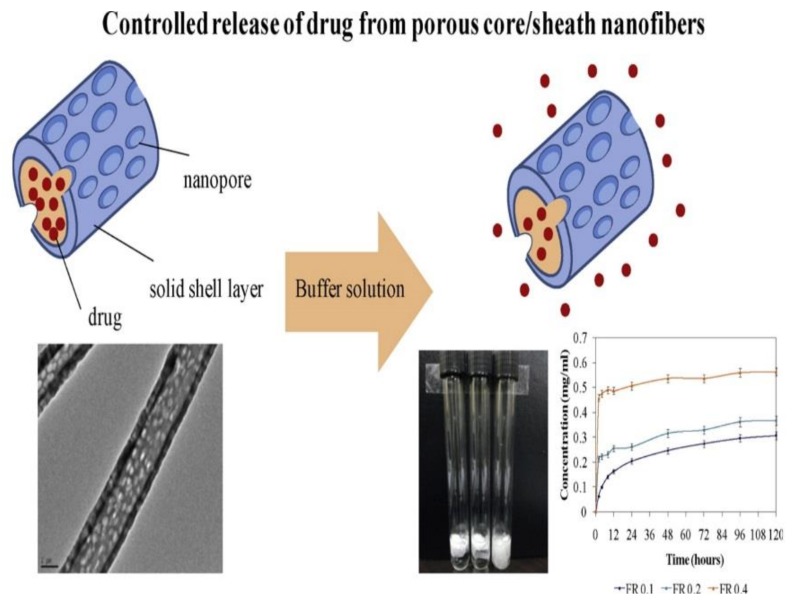
Synthesized porous core/shell composite nanofibers by coaxial electrospinning Reproduced with permission from [84]. Elsevier, 2012.

One of the problems facing the drug delivery system using electrospinning technique is the fast dissolution of the drug on the surface layer of nanofibers, known as burst release or effect. To circumvent this, drugs were coated with monodispersed core/shell particles with the outer layer of polymer. Core/shell composite nanofibers of drugs and bioactive materials can be synthesized using coaxial electrospinning [91,92]. Two miscible or immiscible materials can be electrospun together through this technique, which consisted of two axial capillaries. Using this approach, drugs can be coated by an outer shell of polymeric layer to enhance the release of these materials. Besides, active agents can be protected from the environment by core/shell structure [84]. In 2008, Xie et al. [93] addressed the one-step fabrication of monodispersed drug/poly(lactic-co-glycolic acid) core/shell particles with sizes ranging from 165 nm to 1.2 µm utilizing dual-capillary electrospray. In this study, this system showed that the drug was fully coated, and the initial burst release was also reduced [93]. In 2010, Tiwari et al. [94] discussed the release of hydrophilic drug loaded in a core/shell nanofiber, and the study emphasizes the need to understand the parameters that measure the speed of subsequent release and burst effect. Also, the drug was encapsulated by various layers of different polymers, including poly ɛ-caprolactone (PCL), poly-l-lactide (PLLA), and polyvinyl alcohol (PVA) [94]. In 2011, Vukomanovića et al. [95] reported the use of poly(d,l-lactide-*co*-glycolide)/hydroxyapatite (PLGA/HAp) core/shell structures as clindamycin-base and clindamycin-2-phosphate carriers. Controlled release was observed for the phosphate drug, and sustained release was observed for the base drug. Besides, they realized a high overall content of the released drug during 30 days in vitro. In 2012, Nguyen et al. [84] reported the preparation of porous blended salicylic acid (SA) and polyethylene glycol-poly(lactic acid) core-shell nanofibers (Figure 5). In this work, the core/shell composite nanofibers were obtained with porous and nonporous sheath layers at a core feed rate of 0.1 mL/h. Sustained SA release over five days and high pore density were achieved at this core feed rate. In addition, SA release for porous core/shell nanofibers prepared at this core feed rate was achieved by the Fickian diffusion mechanism. The non-toxicity of these nanofibers was confirmed by using 3T3-L1 and CCD-986sk cells [84].

For biphasic drug release, Jiang et al. prepared core/shell nanofibers by using coaxial electrospinning of Zein (core), polyvinylpyrrolidone (shell), and ketoprofen (KET) (drug) [96]. In this study, the nanofibers were synthesized with an average diameter of 730 ± 190 nm and core thickness of ca. 90 nm. In addition, immediate KET release of 42.3% and sustained release over 10 h were investigated from in vitro dissolution measurements [96].

**Figure 6:**
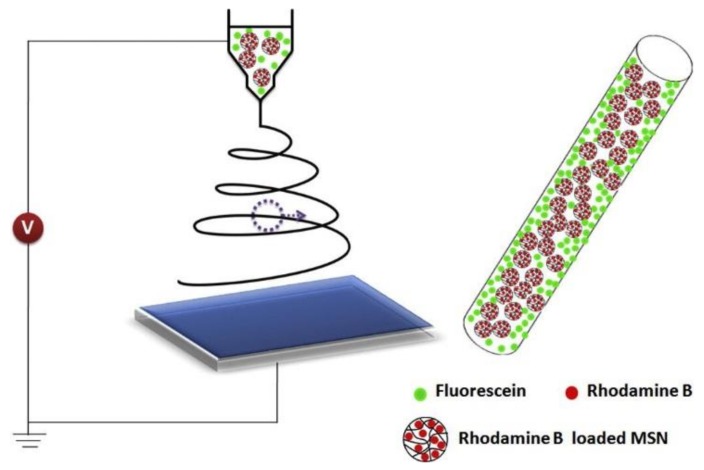
An illustrative diagram of the preparation procedure of dual drug-loaded fibers and the location of the two drugs sited in the composite fibers. Reproduced with permission from [97]. Elsevier, 2012.

Moreover, Song et al. [97] used electrospinning to prepare dual drug (rhodamine B (RHB) and fluorescein (FLU)) poly(lactic-*co*-glycolic acid) (PLGA)/mesoporous silica nanoparticles (MSNs) composite mat, as clearly shown in Figure 6. It was found that RHB offered a sustained release performance owing to interruption from MSNs and PLGA network, while FLU released at a faster rate, as is cited in the polymer galleries [97]. Loh et al. prepared a new class of thermosensitive hydrogel nanofibers based on poly(propylene glycol) (PPG), PCL, and PEG for temperature-controlled protein release [98]. These hybrid nanofibers displayed a remarkable enhancement over common PCL nanofibers. In addition, these nanofibers improved the cell growth and adhesion [98].

There are major requirements of drug release scaffolds such as effective drug control, biocompatibility, and biodegradability. [99]. Towards this end, different strategies are applied such as pH-responsive nanofibers [100], in situ cross-linking of poly(N-isopropylacrylamide) (PNIPAM) with gelatin nanofibers [101], egg albumin-PVA nanofibers [102], CO_2_-infused nanofibers [103], core-shell nanofibers with monolithic core [104], hyperthermia nanofibers [105], and cross-linked cellulose nanofibers [106]. These techniques enhanced the drug loading, cell attachment, and mass transfer performance, thus controlling the drug pharmacokinetics and drug diffusion from the inside of nanofibers into the outside environment. Recently, Mu et al. addressed the incorporation of *cis*-diamminediiodoplatinum (*cis*-DIDP) into PCL nanofibers to enhance the toxicity, water solubility, and cross-resistance properties [107]. The drug loading, drug release, and anti-cancer performance were examined in vitro. Besides, *cis*-DIDP sustained-releases from PCL nanofibers inhibited lung tumor cells in vitro. It was realized that DIDP@PCL are promising controlled drug release systems. This approach was used to develop anticancer chemotherapy [107].

#### 2.1.4. Tissue Engineering

The fabrication of promising matrices/scaffolds that mimic the biological function and structure of the natural extracellular matrix is one of the essential contests in the research of biomaterials and tissue engineering. The smaller human cells can attach to the fibers with dimensions smaller than those of the human cells [1,108]. Hence, nanofibers can offer a promising structure for cells to grow, migrate, and seed. The development of nanofibers for cell proliferation and adhesion is necessary for the organ and tissue regeneration. For this approach, it is essential to reproduce and create a three-dimensional biocompatible composite for cell growth for tissue replacement and repair processes. Great attention has been given to design and develop scaffolds with biodegradable polymer and/or synthetic biopolymer nanofibers [1,109,110,111]. In general, nanofiber-based biopolymers are an excellent candidate with which to mimic native structures compared to large diameter fibers, which do not mimic the structural properties of the native fibrils [1].

##### Blood Vessels

One of the critical tasks in tissue engineering is the design of blood vessels, which carry out the vital task of delivering blood to and from the heart. It is well known that scientists have to deal with a patient’s blood vessel during a transplant, since most of the current synthetic scaffolds are not suitable for use. This process consumes time and increases the required time for a patient’s recovery [78]. Therefore, considerable attention has been devoted to developing blood vessel substitutes. There are many electrospinning processes that are used to design artificial blood vessel scaffolds [78]. Electrospinning process was widely employed to fabricate tubular scaffolds of different lengths and diameters of natural and synthetic polymers for vascular grafts [112,113,114]. The tubular scaffolds can be designed to be strong and flexible, so that they look like blood vessels [112]. Electrospun nanofibers offer high connective pores for cell nutrients exchange [112]. Different synthetic polymers with vascular proteins were used to prepare electrospun tubular scaffolds with small diameters [113,115].

**Figure 7:**
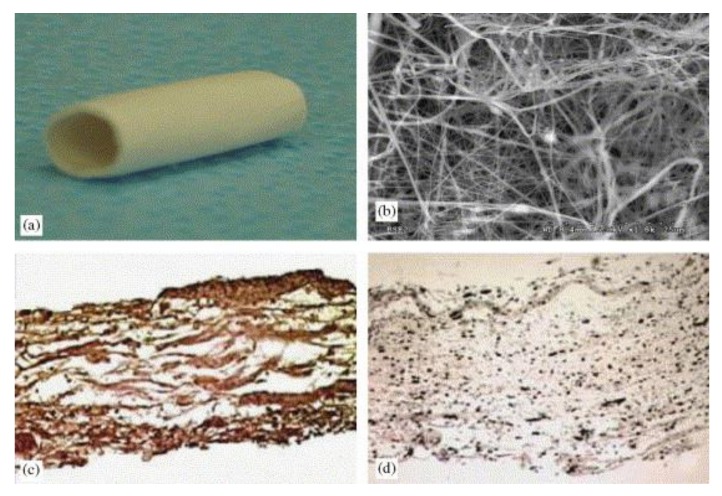
(**a**) Scaffold before crosslinking; (**b**) scaffold before crosslinking at a magnification power of 1800×; (**c**) immunohistochemical analysis utilizing antibodies specific to collagen type I; (**d**) scaffold with 15% elastin shown a homogenous elastin network. Reproduced with permission from [119]. Elsevier, 2006.

Drilling et al. [116] employed PCL to synthesize a burst pressure-competent and suture retention strength (SRS) vascular graft via electrospinning. Tubes were prepared and offered SRS values matching relevant natural tissue and average burst pressure up to 4000 mmHg (more than the standard of 2000 mmHg). Pektok et al. [117] discussed the preparation of PCL-based grafts with better endothelialization and healing properties in vivo than expanded polytetrafluoroethylene (e-PTFE). They showed that faster extracellular matrix formation was achieved with the decomposition of nanofibers grafts. Consequently, these nanofibers with excellent healing properties can be applied to revascularization processes. Also, Huang et al. [118] reported the fabrication of Type I collagen-PEO fibers by electrospinning the purified collagen solution in ambient conditions. In this research, nanofibers with a diameter of 100–150 nm were obtained. The study also showed that elastic modulus and tensile strength values of the prepared non-woven fibers were controlled by the weight ratio of the collagen-PEO composites. In addition to a weight ratio of 1:1, the nanofibers exhibited promising mechanical characteristics due to enlarging the intermolecular bonding between collagen and PEO materials [118]. Stitzel et al. [119] fabricated tubular scaffolds based on polyglycolic acid, collagen, and elastin with characters similar to the native arteries. The fibers were prepared with a thickness of 1 mm and a length of 12 cm. Figure 7 demonstrates a representative 2 cm blend.

Wang et al. reported the fabrication of vascular grafts from PEG and PU mixtures using the blending method. The electrospinning technique-prepared scaffolds were characterized by high porosity and random nanofibrous structure. In this work, the effect of PEG content on vascular diameter and hydrophilicity has been studied and it is reported that with increasing the amounts of PEG, the hydrophilicity of the prepared scaffolds was enhanced remarkably. In contrast, the nanofibers’ diameter decreased with enlarging of the PEG loading. In addition to that, the tensile characteristics of PU/PEG nanofibers were starkly different from those of the neat PU nanofibers, which resulted from the hardening or plasticizing effect imparted by PEG [113]. Cell proliferation was improved in the direction of fiber orientation [115]. Aligned nanofibers displayed direct guidance to control the micromorphology of the engineered tissues [78,120].

**Figure 8:**
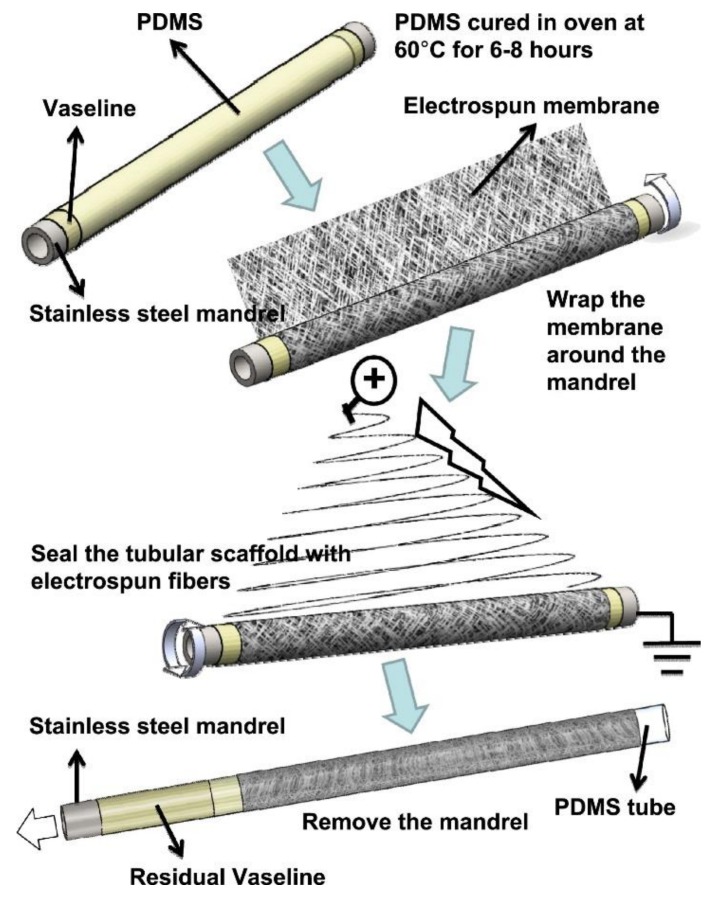
Illustrative scheme of the method of synthesizing the tubular scaffold membranes. Reproduced with permission from [120]. Elsevier, 2012.

Rotating collectors were employed to control the orientation of the nanofibers [78,121]. These collectors can measure the electric fields during the electrospinning process [122]. The resultant network of aligned nanofibers can be then rolled into tubular scaffolds. By controlling the electric field and using rotating tubes, the fibers can be aligned to form tubular scaffolds [78]. On the other hand, Hu et al. [120] fabricated aligned tubular scaffolds similar to arterial media utilizing a scaffold membrane concept (Figure 8). In this approach, fabrication of aligned electrospun fibers has been achieved by collecting poly([ε]-caprolactone) nanofibers on a rotating drum at three various rotation speeds (250, 500, and 1500 rpm). This method fabricated a large-area membrane, which is required for scaffold membranes. The membranes were then enveloped around a mandrel to produce the tubular scaffolds. Mechanical testing can be achieved with aligned fibers at specific directions. Histology demonstrated that cells aligned to the fiber directions demonstrate the approach of measuring the TEVGs micromorphology utilizing these scaffold membranes [120].

To mimic the native ECM, Vatankhah et al. used a blend ratio of 30:70 gelatine:tecophilic to prepare a unique electrospun nanofiber that improved the contractility of smooth muscle cells (SMCs), and then studied the blood compatibility and biochemical characteristics [123]. The fabricated nanofibers were noted to exhibit high mechanical durability, compliance and burst strength, and blood-compatible surface to mimic the antithrombotic surface of the native intima.

Cardiovascular diseases have become the leading cause of death around the globe [124]. These diseases involve inflammatory, infraction, failure, coronary, and stroke. In this respect, electrospun nanofibers can be employed as biodegradable vascular grafts [125]. Of these, Bertram et al. [126] prepared PCL-based nanofibers integrated with collagen for vascular tissue applications. Recently, Abdal-hay et al. electrospun polyurethane onto an airbrushed tube made of PCL to fabricate tubular scaffolds with improved mechanical performance for vascular tissue engineering [127].

##### Bone

Extracellular matrices (ECMs) of hard tissues are used to prepare a nanoscale composite between the organic and inorganic constituents. Hydroxyapatite (HA) (calcium phosphate mineral) nanocrystals are the main component of the composite’s inorganic fraction, which are found in the collagen fibers (Type 1), which represent approximately 95% of the network. The rest of the organic components depend on the tissues (dentin, bone, and enamel). Fibrous collagen protein produces the major morphological matrix [128]. Mechanically, the organic matrix offers elasticity, while the inorganic crystals provide hardness, thereby contributing to a tough and strong ECM [125]. It is noteworthy to mention that bone acts as a storage candidate for phosphate and calcium. Compact (cortical/dense) and spongy (medullary, trabecular, and cancellous) bones are the two types of mature bones [78].

Tissue-engineered bone grafts can treat defects in bones due to trauma such as bone infection, fractures with bone loss, or bone tumors. A bone graft is defined as a small piece of bone relocated to another area of the skeleton to enhance the strength and function of that area. Also, a bone graft is gathered from a corpse, but often it is taken from the same person for whom it will be employed.

The calcified morphology and the calcification technique are critical qualities of hard tissues. Consequently, great effort is devoted to the artificial network that simplifies the calcification performance. The application of bioactive ceramics such as calcium phosphate glasses and crystals with bioactive components has facilitated the in vivo and in vitro calcification techniques by being included in the hard tissue formation. In this approach, bioactive inorganics with biopolymers are considered to be favorable materials in the development of artificial matrices for the regeneration of hard tissue [129].

Yu et al. [130] synthesized chitosan/alginate nanofibers encapsulated by AHp and collagen to decrease the collagen solubility at the implanted place. The core-shell morphology with large surface area was attained using electrospinning process. The positive charge distribution on the surface was hindered by using chitosan. The collagen decomposition over a long period of time was measured by the coating using HAp and collagen. The fabricated chitosan/alginate/collagen-HAp nanofibers improved cell spreading, attachment, mineralization, and proliferation. This approach was used to stabilize collagen bioactivity over long period of time for different bone tissue engineering applications [130]. In addition, Boakye et al. [131] electrospun ε-PCL-MgO-keratin composite nanofibers. In this approach, keratin was first separated from human hair and then mixed with MgO and PCL. The nanofibers displayed a positive magnesium release, good tensile characteristics, and excellent fiber structure. It was observed that Young’s modulus and tensile strength decreased with increasing MgO content in the fibers. Also, the addition of keratin increased ultimate tensile strength and Young’s modulus.

Silk fibroin (SF) is a natural polymeric fiber involved in various biomedical applications including bone tissue engineering owing to its biodegradability, flexibility, biocompatibility, and elasticity, but its usage in the orthopedic area is limited because of its weak osteogenic capacity. HA is a biocompatible and bio-ceramic material, and is an appropriate candidate for generating orthopedic and dental substitutes; its usage is limited due to its brittle nature. Consequently, SF and HA were blended to combine their characteristics and to synthesize a unique bone constructing template [132,133]. In this concept, alginate (AL)/HA/SF composites were fabricated by Jo et al. as bone tissue scaffolds for rat calvarial defects [132]. These scaffolds displayed remarkably higher new bone generation with respect to the control. Moreover, the residual graft in AL/HA scaffolds is higher than that in AL/HA/SF scaffolds. The AL/HA/SF scaffolds did not induce giant cell formation or inflammatory reaction because of their excellent biocompatibility properties. This group offered a lower expression of tumor necrosis factor (TNF)-α compared to that of AL and AL/HA groups (*p* < 0.001 and *p* = 0.001, respectively). Additionally, AL/HA/SF scaffolds observed high expression of osteogenic markers such as Runt-related transcription factor (Runx2), osteoprotegerin (OPG), and fibroblast growth factor-23 (FGF-23).

Owing to the ease of preparation, surface functionalization, and pore size tailoring, polymeric nanofibers and bio-ceramic nanoparticles-introduced nanofibers have been applied to improve promoted cell homing activity and dental regeneration [134]. PCL-templated nanohydroxyapatite (tHA) composites were fabricated by Gao et al. as artificial bone extracellular matrix [135]. These composites exhibited better osteogenic performance and promoted the regeneration of lamellar-like bones in a rat calvarial defect. Besides, nanoscale hydroxyapatites were highly dispersed on cellulose nanofibers for bone regeneration by Yamaguchi et al. [136]. The nanofibers improved the proliferation of osteoblastic MC3T3-E1 cells. Recently, Guler et al. [137] fabricated functionalized *ε*-PCL/poly(m-anthranilic acid) nanofibers as efficient scaffolds with desired structural, mechanical, and functional properties for bone tissue applications. Interestingly, boron nitride/gelatin nanofibers were electrospun for bone tissue applications [138]. The electrospun mats were stable after the glutaraldehyde cross-linking, and the fibrous structure was well maintained. Chen at al. [139] electrospun hierarchically ordered polymer nanofiber shish kebabs to perform as bone scaffolds. It was confirmed that the crystallization of PCL-b-poly(acrylic acid) copolymer on PCL nanofibers resulted in a novel shish kebab morphology, remarkably improving the mechanical performance of the nanofibers, which are cytocompatible to L-929 mouse fibroblast cells.

Recently, poly(3-hydroxybutyrate-*co*-4-hydroxybutyrate)/graphene oxide scaffolds were prepared by Zhou et al. for bone repair [140]. It was realized that these scaffolds exhibited a favorable porous morphology, fast osteogenic capability, and improved biomechanical performance [140]. Interestingly, starch-derived nanographene oxide scaffolds were fabricated by Wu et al. [141] for bone tissue engineering. The biodegradable scaffolds underwent simultaneous mineralization process and degradation during 1 week of mineralization test and cell culture, thus mimicking the function and morphology of ECMs for potential bone tissue applications [141]. Qian et al. [142] fabricated an efficient multi layers scaffold with osteoconductive behavior and antibacterial activity to regenerate bone defects. The main components of the biocompatible multi layers structure are chlorhexidine-doped-PLGA/PCL, PLGA/PCL and β–tricalcium phosphate-doped-PLGA/PCL [142]. The influence of polyacrylonitrile/MoS_2_ composite nanofibers on the growth performance of bone marrow mesenchymal stem cells (BMSCs) was reported by Wu et al. [143]. The composite nanofibers were investigated to develop BMSC contact with each other, improve cellular behavior, and also offer positive promotion to regulate cellular proliferation [143]. For guided spinal fusion, Qu et al. presented an injectable and thermosensitive hydrogel made of collagen/n-HA/BMP-2@PCEC/PECE that enclosed in poly(d,l-lactide) (PDLLA) nanofibrous membranes to provide a barrier between the soft tissue and hydrogels [144]. This system prevented the escaping factor to maintain retention in the desired sites of osteogenesis [144].

Table 1 represents different nanofibers used on medical prostheses, wound dressing, and tissue engineering including blood vessels and bones. From Table 1, it is clearly observed that PCL can be used in all these different biomedical approaches.

## 3. Conclusions and Future Perspective

Throughout this review, new insights for biomedical applications have been addressed, focusing on the promising benefits of employing electrospun nanofibers. It is evident from ineffective conventional fiber preparation techniques that there is a desperate need for a distinct and unique fabrication method. As extensively introduced, electrospinning technology has the potential to play a critical function in biomedical applications. Towards this end, the drug can be introduced easily into the nanofibers. In this approach, the challenge is associated with controlling the matrix composition impact and measuring the suitable cell populations. Changing the wall thickness of the fabricated nanofibers can control the overall drug delivery rate. For tissue-engineering biomedical scaffolds, porous 3-D scaffolds from bioabsorbable polymers can be synthesized. The morphology can support the healing process by offering many cites for cell tissue and cell growth. The fabricated scaffolds do not need later surgical elimination, as they break down naturally within the body and the normal metabolic pathways dispose of the by-products. To sum up, electrospun nanofibers are considered to be promising candidates for biomedical applications due to their flexibility in imparting unique medical properties and high continuous production rates. However, in vivo stability and more detailed toxicity studies should be covered in the future for the electrospun nanofibers to be clinically approved.

## Figures and Tables

**Table 1:** Summary for different nanofibers used on various biomedical applications.

Application		Fibrous Material	Reference
Medical prostheses		Copolymer of ε–caprolactam and hexamethylendiaminadipate	Popryadukhin et al. [32].
		Polycaprolactone (PCL)/chitosan (CS)	Semnani et al. [33]
Wound dressing		Polyurethane (PU)/dextran	Unnithan et al. [34]
		Alginate/chitin	Jayakumar et al. [45,51]
		Dibutyrylchitin (DBC)	Chilarski et al. [46]
		Carboxyethyl chitosan/poly(vinyl alcohol) (CECS/PVA)	Zhou et al. [48]
		Quaternary chitosan (QCS)	Ignatova et al. [49]
		CS/collagen	Wang et al. [52]
		Polyethylene/chitosan/type 1 collagen	Chen et al. [53]
		Sodium alginate (SA)/PVA	Shalumon et al. [54]
		Silk fibroin/CS	Cai et al. [56]
		PCL/gelatin	Chong et al. [57]
		PVA/CS-ethylenediaminetetraacetic acid (CS-EDTA)	Charernsriwilaiwat et al. [59]
		N-heterocyclic carbene complexes	Elzatahry et al. [60]
		Poly(lactic acid)(PLA)/CS	Li et al. [61]
		Collagen/tropoelastin	Rnjak-Kovacina et al. [62]
		Nylon-6/Ag nanoparticles (Ag NPs)	Pant et al. [63]
		ZnO/Ag/PU	Shamshi Hassan et al. [64]
		Collagen/Ag NPs	Rath et al. [66]
		Poly(ethylene oxide) (PEO)/PCL/Ag NPs	Dubey et al. [67]
		CS/PEO/green tea extract	Sadri et al. [68]
		Gelatin/vitamins A and E	Li et al. [69]
		Honey/CS/Cleome droserifolia and Allium sativum	Sarhan et al. [70]
		PVA/CS/tetracycline hydrochloride	Alavarse et al. [71]
		PCL/Levofloxacin	Pásztor et al. [72]
		Dimethyloxalylglycine-embedded PCL	Zhang et al. [74]
		Collagen-coated ostholamide	Kandhasamy et al. [75]
		Silk fibroin (SF)/graphene oxide (GO)	Wang et al. [76]
Drug release		PLA/ poly(ethylene-co-vinyl acetate) (PEVA)/tetracycline hydrochloride	Kenawy et al. [86]
		Hydroxylpropylmethyl cellulose (HPMC)/Itraconazole	Verreck et al. [87]
		Poly(d,l-lactide-*co*-glycolide)/hydroxyapatite (PLGA/HAp)/clindamycin-base and clindamycin-2-phosphate	Vukomanovića et al. [95]
		Zein/polyvinylpyrrolidone/ketoprofen (KET)	Jiang et al. [96]
		Rhodamine B (RHB) and fluorescein (FLU)/poly(lactic-*co*-glycolic acid) (PLGA)/mesoporous silica NPs (MSNPs)	Song et al. [97]
		Poly(4-vinylbenzoic acid-*co*-(*ar*-vinylbenzyl)trimethylammonium chloride) [poly(VBA-*co*-VBTAC)]/ciprofloxacin	Demirci et al. [100]
		Poly(*N*-isopropylacrylamide) (PNIPAM)/gelatin	Slemming-Adamsen et al. [101]
		Egg albumin/PVA	Zahedi et al. [102]
		PCL/PCL–gelatin/BODIPY 493/503 and Rhodamine B fluorescent	Geiger et al. [103]
		Poly(methyl methacrylate) (PMMA)/PVA/ciprofloxacin hydrochloride (CIP)	Zupančič et al. [104]
		Oxidized cellulose/branched polyethyleneimine (bPEI)/amoxicillin (AM) and ibuprofen (IB)	Fiorati et al. [106]
		*cis*-diamminediiodoplatinum (*cis*-DIDP)/PCL	Mu et al. [107]
Tissue engineering	Blood vessels	PEG/PU	Wang et al. [113]
		PCL	Drilling et al. [116] and Hu et al. [120]
		PCL and polytetrafluoroethylene (e-PTFE)	Pektok et al. [117]
		Type I collagen-PEO	Huang et al. [118]
		Polyglycolic acid/collagen/elastin	Stitzel et al. [119]
		Gelatin/tecophilic	Vatankhah et al. [123]
		Collagen/PCL	Bertram et al. [126]
		PU/PCL	Abdal-hay et al. [127]
	Bone	CS/alginate/AHp/collagen	Yu et al. [130]
		PCL/MgO/keratin	Boakye et al. [131]
		PCL/HA	Gao et al. [135]
		HA/cellulose	Yamaguchi et al. [136]
		PCL/poly(m-anthranilic acid)	Guler et al. [137]
		Boron nitride/gelatin	Nagarajan et al. [138]
		PCL-b-poly(acrylic acid) copolymer/PCL	Chen at al. [139]
		Poly(3-hydroxybutyrate-*co*-4-hydroxybutyrate)/graphene oxide	Zhou et al. [140]
		Starch/GO	Wu et al. [141]
		Chlorhexidine-doped-PLGA/PCL, PLGA/PCL and β–tricalcium phosphate-doped-PLGA/PCL	Qian et al. [142]
		Polyacrylonitrile/MoS_2_	Wu et al. [143]
		Poly(d,l-lactide) (PDLLA)	Qu et al. [144]
